# Identification of a serotonin N-acetyltransferase from *Staphylococcus pseudintermedius* ED99

**DOI:** 10.3389/fmicb.2023.1073539

**Published:** 2023-02-22

**Authors:** Nourhane Hafza, Ningna Li, Arif Luqman, Friedrich Götz

**Affiliations:** ^1^Microbial Genetics, Interfaculty Institute of Microbiology and Infection Medicine Tübingen (IMIT), University of Tübingen, Tübingen, Germany; ^2^Cluster of Excellence “Controlling Microbes to Fight Infections”, University of Tübingen, Tübingen, Germany; ^3^Biology Department, Institut Teknologi Sepuluh Nopember, Surabaya, Indonesia

**Keywords:** N-acetyltransferase, N-acetylserotonin, serotonin N-acetyltransferase, neurochemicals, *Staphylococcus* sp., *Staphylococcus pseudintermedius*, microorganisms

## Abstract

Serotonin N-acetyltransferase (SNAT) catalyzes the biosynthesis of N-acetylserotonin (NAS) and N-acetyltryptamine (NAT), two pleiotropic molecules with neurotransmitter functions. Here, we report the identification of a SNAT protein in the genus *Staphylococcus*. The SNAT gene identified in *Staphylococcus pseudintermedius* ED99, namely *SPSE_0802*, encodes a 140 residues-long cytoplasmic protein. The recombinant protein SPSE_0802 was expressed in *E. coli* BL21 and found to acetylate serotonin (SER) and tryptamine (TRY) as well as other trace amines *in vitro*. The production of the neuromodulators NAS and NAT was detected in the cultures of different members of the genus *Staphylococcus* and the role of SPSE_0802 in this production was confirmed in an ED99 *SPSE_0802* deletion mutant. A search for SNAT homologues showed that the enzyme is widely distributed across the genus which correlated with the SNAT activity detected in 22 out of the 40 *Staphylococcus* strains tested. The N-acetylated products of SNAT are precursors for melatonin synthesis and are known to act as neurotransmitters and activate melatonin receptors, among others, inducing various responses in the human body. The identification of SNAT in staphylococci could contribute to a better understanding of the interaction between those human colonizers and the host peripheral nervous system.

## Introduction

Acetylation of biomolecules is a widespread strategy used for regulation of cellular processes such as protein synthesis, detoxification, and virulence. In higher organisms, acetylation also serves to modify small molecules into hormones or neurotransmitters with diverse biological activities (Hu et al., 2010; Hentchel and Escalante-Semerena, 2015). Serotonin N-acetyltransferases (SNAT) belong to the superfamily of GCN5-related N-acetyltransferases (GNAT) which catalyze the acetylation of various molecules. SNAT acetylate monoamines, such as serotonin (5-hydroxytryptamine), tryptamine and other trace amines (TAs), by transfer of an acetyl moiety from acetyl coenzyme A (acetyl-CoA) onto the target substrate (Burckhardt and Escalante-Semerena, 2020).

In mammals, serotonin (SER) is an important hormone and neurotransmitter that regulates a myriad of functions in the CNS, including emotion, cognition and stress. In the enteric nervous system, SER is involved in gastrointestinal motility and secretion (Gershon and Tack, 2007). SER can be modified by SNAT into N-acetylserotonin (NAS), another pleiotropic molecule which, in addition to being the direct precursor of melatonin (N-acetyl-5-methoxytryptamine; Axelrod and Weissbach, 1960), can itself act as an agonist of melatonin receptors MT3, which are predominant in the gut (Jockers et al., 2016) and thereby modulate the circadian rhythm. The amounts of NAS in the brain and the gut are critical for regulating the melatonin biosynthesis pathway (Brown et al., 1983, 1984; Klein, 2007; Maronde et al., 2011). Independently from the melatonin pathway and receptors, NAS also performs potent anti-depressant, neurotrophic and cognition-enhancing effects (Nosjean et al., 2000; Oxenkrug and Ratner, 2012; Tosini et al., 2012). Those effects are shown to be the result of NAS’ robust activation of the tyrosine kinase receptor TrkB, presumably by direct binding (Jang et al., 2010).This activation is essential in regulating hippocampal neurogenesis and sensitivity to anti-depressive drugs (Li et al., 2008). In addition to receptor-mediated signaling, NAS possesses important anti-oxidant (Wölfler et al., 1999; Oxenkrug, 2005) and immune-modulating effects (Lotufo et al., 2001; Perianayagam et al., 2005).

Tryptamine (TRY) is a so-called trace amine (TA) that despite being present in trace amounts in the brain, is no less important than SER. TRY can act directly on SER receptors and modulate the effects of the latter (Jones, 1982). Its acetylated form, N-acetyltryptamine (NAT), is similarly to NAS, a partial agonist of melatonin receptors (Dubocovich, 1995).

SER, TRY, and their acetylated forms (NAS and NAT) perform important functions in many organisms *via* receptor-mediated or receptor-independent signaling pathways. Hence, they are highly conserved and their synthesis is tightly regulated.

Gram-positive bacteria are thought to be the evolutionary origin of melatonin in vertebrates (Iyer et al., 2004; Coon and Klein, 2006), which suggests that they harbor enzymes involved in melatonin biosynthesis. However, only little is known about enzymes involved in melatonin biosynthesis in these microorganisms. Interestingly, we recently identified the enzyme ‘staphylococcal aromatic amino acid decarboxylase’ (SadA) responsible for SER and TRY production in several staphylococcal species. In *Staphylococcus pseudintermedius* ED99, SadA is a promiscuous enzyme decarboxylating all biogenic aromatic amino acids such as 5-hydroxytryptophan (5-HTP) and tryptophan into SER and TRY, respectively (Luqman et al., 2018). SadA products perform different functions and are found to be beneficial for both the producing bacteria and the host. For instance, they promote the adherence and internalization of staphylococci into intestinal epithelial cells by activation of α2-adrenergic receptors. As a result, bacterial colonization and survival in the host is increased (Luqman et al., 2018). In contrast, SadA products play a role in accelerating wound healing by acting as β2-adrenergic receptor inhibitors. This inhibitory activity abrogates the negative effect of epinephrine on cell motility and wound healing (Luqman et al., 2020b).

Based on these findings, we aimed to investigate whether staphylococci produce SNAT, the protein responsible for the next step in melatonin biosynthesis, namely the N-acetylation of SadA products. Here, we identified three putative serotonin N-acetyltransferase proteins from *S. pseudintermedius* ED99 (annotated SPSE_0436, SPSE_0802 and SPSE_1761) based on similarity to the cyanobacterium *Synechocystis* sp. SNAT (cSNAT; Byeon et al., 2013). Among the three proteins, SPSE_0802, sharing 39% similarity with the functional cSNAT, exhibited N-acetyltransferase activity *in vitro*, catalyzing the conversion of SER and TRY to NAS and NAT, respectively. The biosynthesis of NAS and NAT was confirmed in *S. pseudintermedius* ED99 cells, as well as in other staphylococcal species. The presence of SPSE_0802 protein homologues in other staphylococci correlated with the ability of the species to produce the N-acetylated monoamines, which confirms the role of SPSE_0802 in NAS and NAT production.

## Results

### Putative SNAT proteins in *Staphylococcus pseudintermedius* ED99 genome

We searched for SNAT homologues in *S. pseudintermedius* ED99, as a model of SER-producing staphylococci using cSNAT identified in the cyanobacterium *Synechocystis* sp. PCC 6803 (GenBank accession no. NP_442603) as a reference. cSNAT is a 171 amino acids long protein possessing an N-terminal sequence comprising at least 22 amino acid residues that have no effect on its enzymatic activity (Byeon et al., 2013). The inactive N-terminal domain is typically found in SNATs in higher organisms and usually serves as a translocation signal.

RefSeq database search was performed to identify homologues in ED99 using BLASTP (O’Leary et al., 2016). The three protein hits that were identified and annotated as SPSE_0802, SPSE_0436 and SPSE_1761 are 140, 138 and 142 amino acids long, respectively. SPSE_0802, SPSE0_436 and SPSE_1761 are annotated as GNAT (GCN5-related N-acetyltransferases) family peptides and they share 19, 18, and 10% identity (34, 33, and 16% similarity) with cSNAT, respectively (Table 1). Comparison to the fully functional truncated cSNAT_23-171_ increases the similarity to 39, 38, and 19% to each of SPSE_0802, SPSE0_436 and SPSE_1761, respectively.

**Table 1:** List of putative SNAT proteins in *S. pseudintermedius* ED99 genome.

Locus tag	Predicted gene product	Protein ID	Protein size (kDa)	Percent identity to cSNAT	Percent similarity to cSNAT
SPSE 0802	Acetyltransferase, GNAT family	ADX76117.1	15.9	19	34
SPSE 0436	Acetyltransferase, GNAT family	ADX75773.1	15.3	18	33
SPSE 1761	Acetyltransferase, GNAT family	ADX77015.1	16.4	10	16

The percent identity and similarity of the putative SNAT proteins to each of the cyanobacterium *Synechocystis* sp. PCC 6803 SNAT (cSNAT) are shown. The percent similarity of SPSE0802, SPSE0436 and SPSE1761 to the fully functional truncated cSNAT_23-171_ (Byeon et al., 2013) is 39, 38, and 19%, respectively.

**Figure 1:**
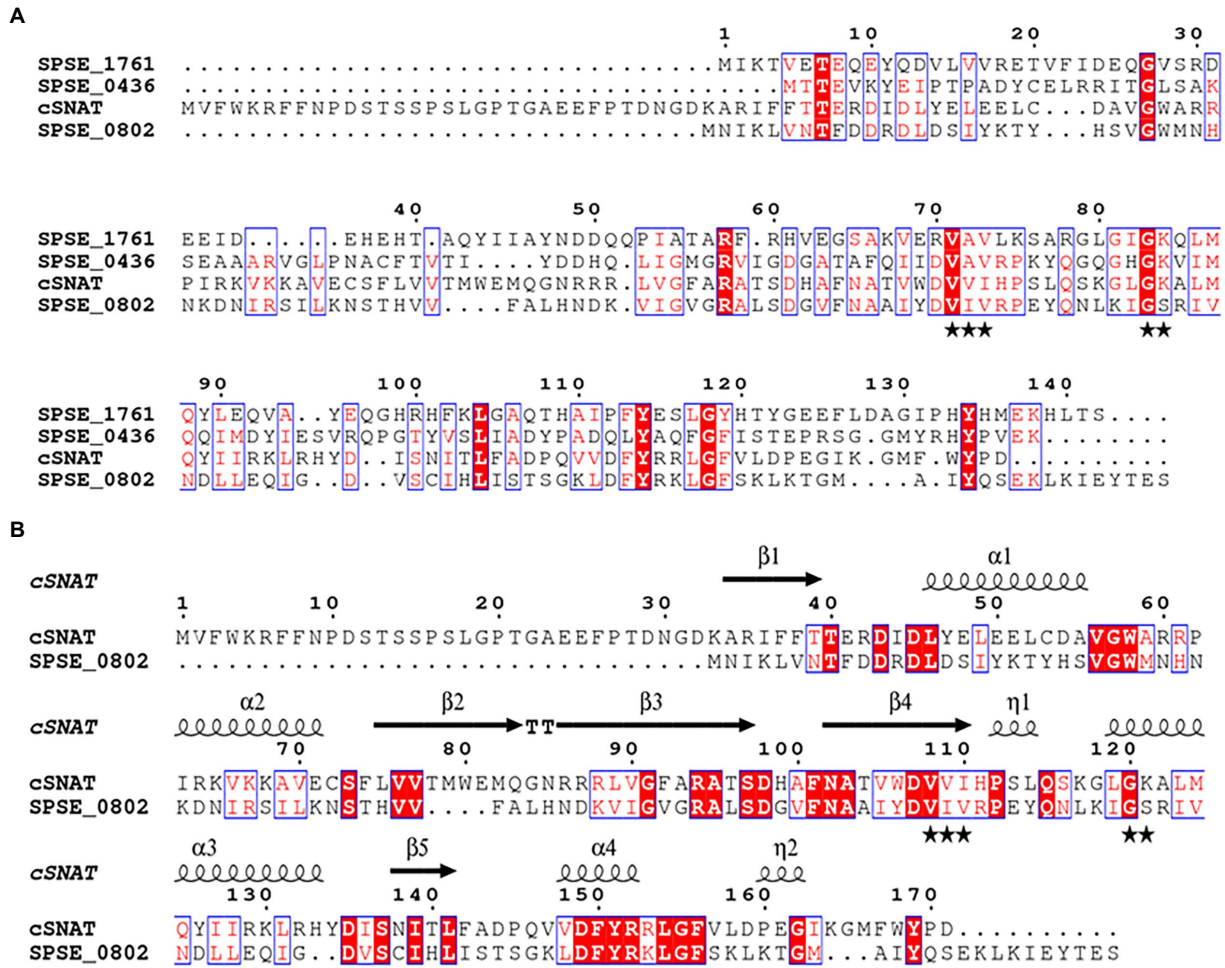
Alignment of the predicted SNAT proteins in *S. pseudintermedius* ED99 genome. (**A**) Protein alignment of the candidate SNATs (SPSE_0802, SPSE_0436 and SPSE_1761) with cSNAT. The acetyl coenzyme A binding pocket indicated with black stars is conserved among the 4 proteins. cSNAT possess an N-terminal sequence comprising at least 22 amino acid residues that have no effect on its enzymatic activity. (**B**) Alignment of cSNAT and SPSE_0802 sharing the highest similarity (34%). 3-D structure prediction shows that both proteins share a common fold known as the GNAT fold, comprised of 6–7 antiparallel β -strands (5 present in cSNAT and SPSE_0802) and 4 α -helices in the topology β1- α1- α2- β2- β3- β4- α3- β5- α4- β6- β7. Amino acid alignment of the proteins was generated with Clustal Omega and structural information (β-sheets and α-helices) was added with ESPript 3.0 based on cSNAT modeling by Swiss Model. Identical residues are shaded red with white letters, and residues with similar properties are in red font surrounded by blue boxes. Black stars underneath the alignment indicate residues forming the coenzyme A binding pocket.

Secondary structure prediction of the three proteins suggests that they do possess neither a signal peptide nor a transmembrane domain. Therefore, the enzymes were predicted to be localized in the cytoplasm similarly to previously identified SNATs. Tertiary structure prediction shows the presence of a conserved acetyl-CoA binding domain in all of the four aligned proteins (Figure 1A). SPSE_0802 shares notable structural similarity with cSNAT presented by a common fold, comprised of 6–7 antiparallel β -strands (5 present in cSNAT and SPSE_0802) and 4 α -helices in the topology β 1- α 1- α 2- β 2- β 3- β 4- α 3- β 5- α 4- β 6- β 7 (Figure 1B).

### *In vitro* enzymatic activity of recombinant SPSE_1761, SPSE_0802 and SPSE_0436

**Figure 2:**
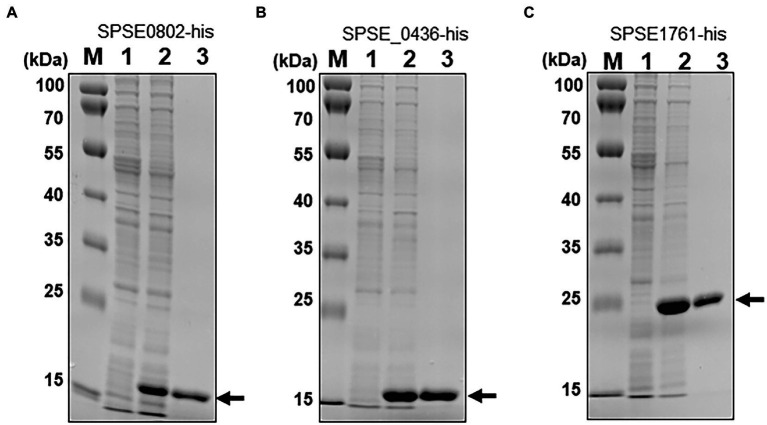
Expression of the *S. pseudintermedius* ED99 putative SNAT genes in *E. coli* BL21 harboring (**A**) SPSE_0802, (**B**) SPSE_0436 or (**C**) SPSE_1761 in the vector pET28a. M, molecular marker; Lane 1, total protein in 10-μL aliquots of bacterial cells without IPTG; Lane 2, total cytoplasmic protein after IPTG treatment; Lane 3, 5 μg soluble protein purified by affinity chromatography. Proteins were separated by 14% SDS-PAGE and stained with Coomassie blue. The recombinant proteins SPSE_0802 and SPSE_0436 migrated to roughly their theoretical sizes (15.9 and 15.3 kDa respectively), whereas SPSE_1761 migrates slightly higher than its theoretical size on the gel.

To determine whether any of the three identified proteins from ED99 are SNAT enzymes, the corresponding genes were cloned into the bacterial expression vector pET28a and expressed as C-terminal His-tagged fusion proteins in *E. coli* BL21. His-tagged SPSE_0802, SPSE_0436 and SPSE_1761 proteins were isolated from the cytoplasmic fraction of *E. coli* BL21 clones and purified by Ni-NTA superflow resin (Figure 2).

**Figure 3:**
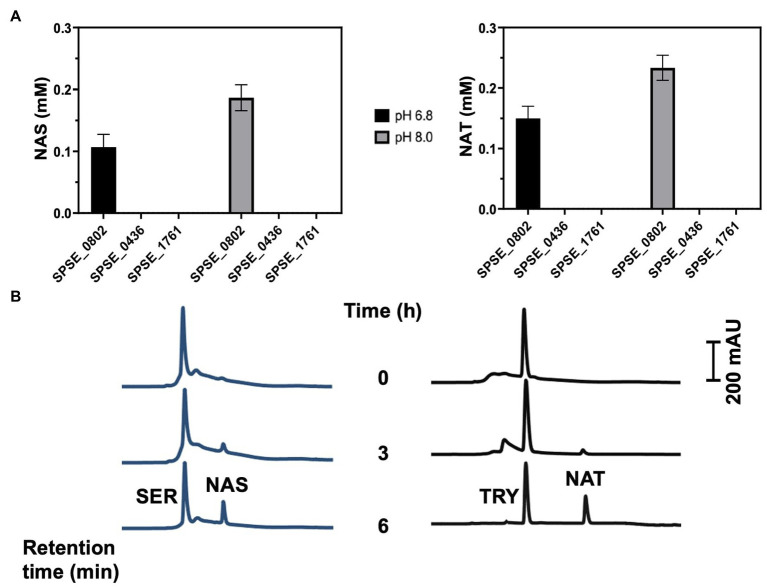
*In-vitro* SNAT enzymatic activity of the recombinant proteins SPSE_1761, SPSE_0802 and SPSE_0436. (**A**) The purified recombinant proteins (200 μg) were incubated in a total volume of 1 mL containing 1 mM serotonin or tryptamine and 1 mM acetyl-CoA in 50 mM potassium phosphate (pH 6.8 or 8.0) at 37°C. The amounts of N-acetylated compounds (NA-SER and NA-TRY) were quantified after 6 hr incubation using HPLC analysis using the standard curve. Only SPSE_0802 could acetylate the substrates. (**B**) Chromatograms of N-acetylation of serotonin and tryptamine by SPSE_0802 after 6-hr incubation in 50 mM potassium phosphate (pH 8.0) at 37°C.

The enzymatic activity of the recombinant proteins was assayed by measuring the N-acetylation of 1 mM SER or tryptamine in presence of 1 mM acetyl-CoA as co-factor at 37°C. SPSE_0802 could acetylate SER and TRY into NAS and NAT, respectively as detected by high performance liquid chromatography (HPLC) analysis (Figures 3A,B). At pH 8.0, SPSE_0802 acetylated around 18% of the total SER and 23% of the total tryptamine after 6 h incubation. At pH 6.8, a lower activity was detected, as only 12% of SER and 16% of tryptamine were acetylated after the same incubation period. In both conditions, however, SPSE_0802 had a slightly higher activity when TRY was used as a substrate. These results match the higher activity at pH 8.0–9.0 and the higher specificity using TRY as substrate reported for other SNATs (Byeon et al., 2013; Kang et al., 2013; Lee et al., 2014). HPLC analysis of the enzymatic reactions using recombinant SPSE_1761 and SPSE_0436 under the same conditions did not detect any N-acetylated products, indicating that neither of these two proteins is a functional SNAT enzyme (Figure 3A).

**Figure 4:**
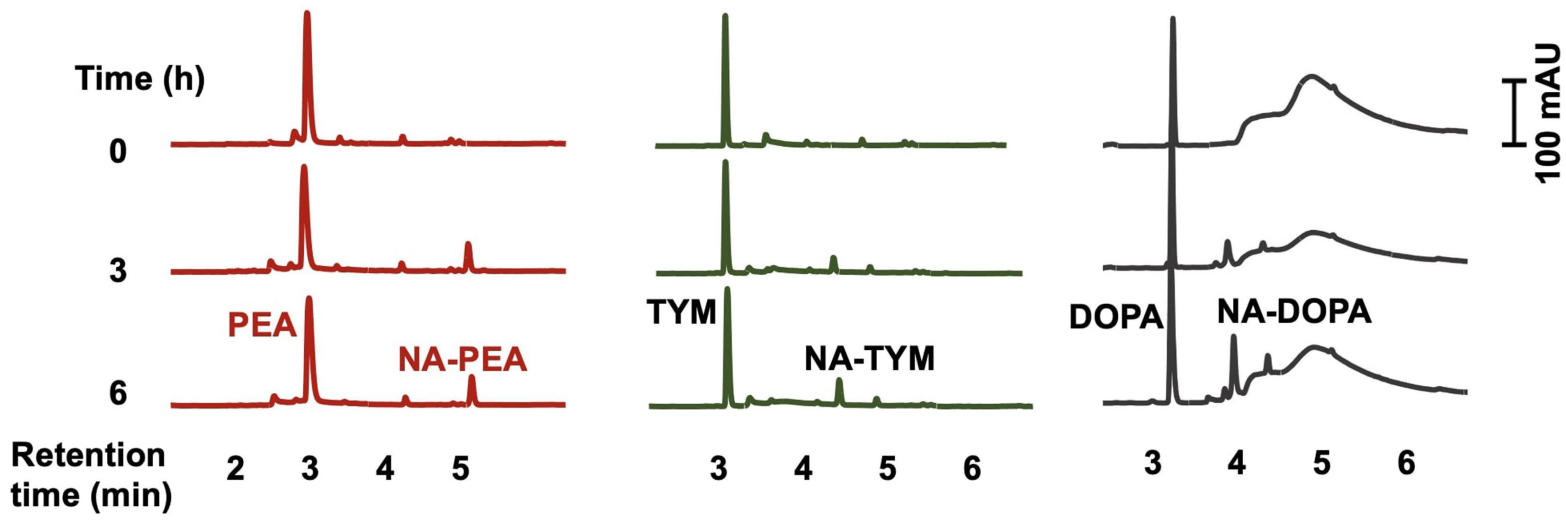
HPLC analysis of *in-vitro* enzymatic activity of SPSE_0802 (50 μg) using 0.5 mM phenethylamine (PEA), tyramine (TYM) and dopamine (DOPA) as substrates after 6-hours incubation in phosphate buffer (pH 8.0) at 37°C. All 3 substrates could be acetylated by SPSE_0802.

SPSE_0802 could also acetylate other substrates such as dopamine, phenethylamine, and tyramine (Figure 4), which shows the wide spectrum of substrate specificity of the enzyme. There was no acetylation of SER and TRY in absence of SPSE_0802, which rules out the possibility of non-enzymatic acetylation of the compounds. To further confirm the activity of SPSE_0802, the recombinant enzyme was inactivated by incubation at 95°C for 10 min before being added to the reaction mixture. In this case, neither NAS nor NAT were detected by HPLC analysis of the reaction after 6 h.

The N-acetylation reaction catalyzed by SPSE_0802 was acetyl-CoA dependent as no activity was seen in the absence of acetyl-CoA. The reaction was also time-dependent, as more acetylated products accumulated after a longer incubation time. The acetylated products were quantified using the standard curves (Supplementary Figure S1) and confirmed by comparing their retention time to synthetic standards in HPLC.

### SNAT activity in *Staphylococcus pseudintermedius* ED99 WT and *SPSE_0802* deletion mutant

To investigate the role of SPSE_0802 in N-acetylation of monoamines in *S. pseudintermedius* ED99, we constructed the deletion mutant ED99Δ*SPSE_0802* and compared both strains’ ability to synthesize NAS. Due to its hydrophilic properties, exogenous SER needs to be actively transported into the cell cytoplasm in order to be processed by SPSE_0802. Since ED99 cannot uptake SER from the extracellular medium, we used 5-HTP, the substrate of SadA decarboxylation and precursor of SER. We have previously shown that the decarboxylation reaction by SadA takes place in the cytoplasm where SER is produced (Luqman et al., 2018). Accordingly, we expected 5-HTP to be processed into NAS in a two-step process: decarboxylation to SER by SadA, followed by N-acetylation to NAS by SPSE_0802.

**Figure 5:**
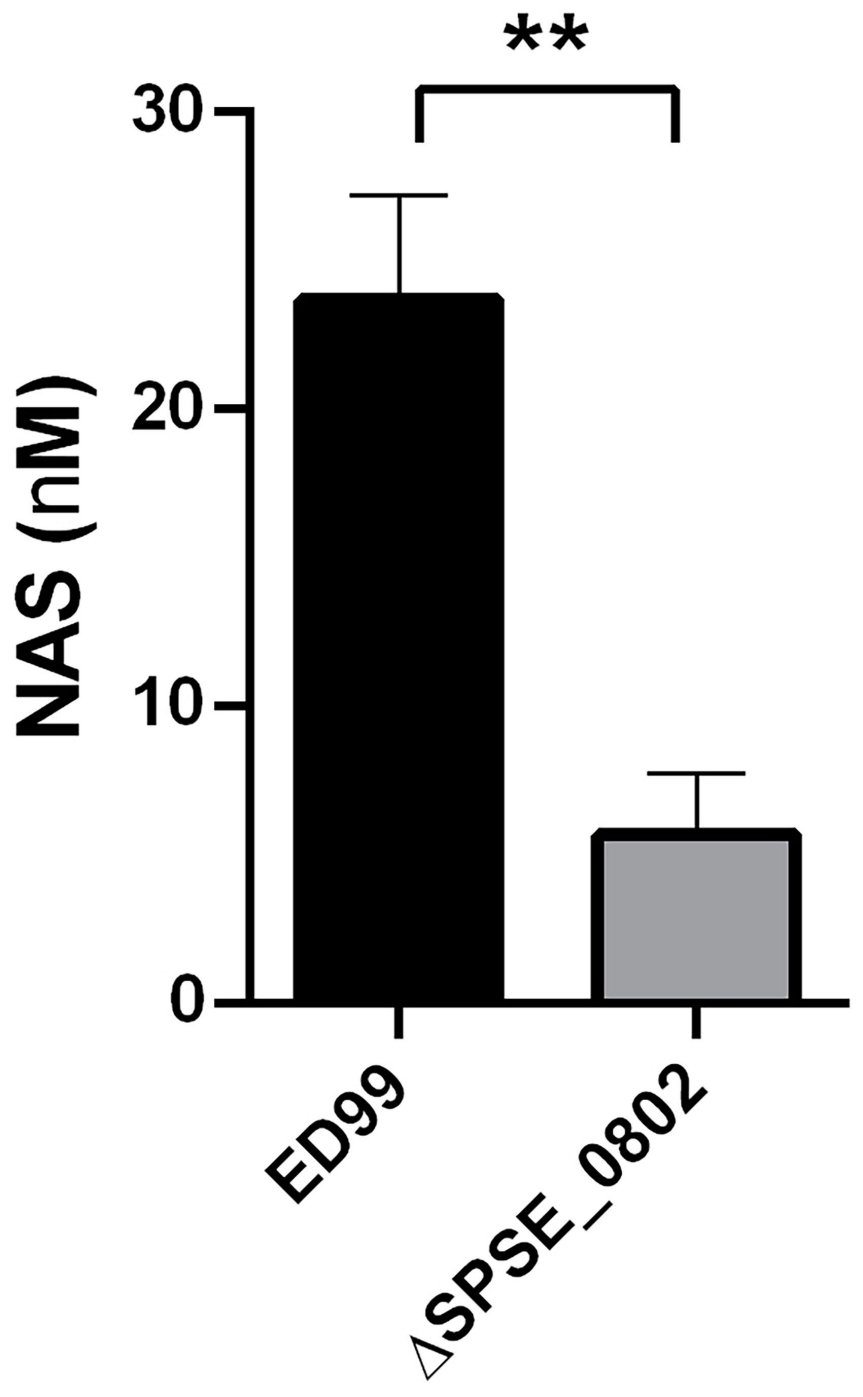
NAS biosynthesis in *S. pseudintermedius* ED99. Quantfifcation of NAS using HPLC analysis from cell lysate of *S. pseudintermedius* ED99 and its congenic SPSE_0802 mutant. The cells were cultured overnight in TSB supplemented with 5 mM 5-HTP as a precursor for NAS production in two reactions; decarboxylation to SER by SadA followed by N-acetylation of SER by SPSE_0802. The cells were incubated for 14 hrs at 37°C, with shaking at 150 rpm. The cells were pelleted and lysed. The cell lysate was collected and stored at -20°C prior to HPLC analysis. Data was analyzed by Mann-Whitney test, **p < 0.01.

*S. pseudintermedius ED99* WT and Δ*SPSE_0802* cells were fed with 5 mM 5-HTP and incubated overnight at 37°C. HPLC analysis of the culture supernatant of cells fed with 5-HTP shows that ED99 WT produced around 24 nM NAS whereas Δ*SPSE_0802* mutant produced around 4 nM NAS (Figure 5). These results verify that ED99 can produce NAS and that SPSE_0802 plays an important role in this production. The residual amounts of NAS in the cultures of the ED99 Δ*SPSE_0802* mutant suggest the presence of another mechanism or enzyme that could produce NAS with a much lower substrate specificity than SPSE_0802.

SNAT enzymes in plants were recently found to be implicated in an alternative pathway in which they catalyze the conversion of 5-methoxytryptamine (5-MT) directly to melatonin (Back et al., 2016; Tan et al., 2016). To test whether the identified SPSE_0802 could also catalyze this reaction in staphylococci, we searched for melatonin production by *S. pseudintermedius* ED99 and its congenic deletion mutant ED99Δ*SPSE_0802*. No melatonin was detected by HPLC analysis of the cells from overnight cultures fed with 5-MT which indicates that SPSE_0802, unlike plant SNAT, cannot use 5-MT as a substrate and that ED99 does not harbor the enzyme involved in the last step of melatonin biosynthesis from N-acetylserotonin (namely, the acetylserotonin o-methyltransferase ASMT) (results not shown).

### SNAT activity in other staphylococcal species

To verify whether other staphylococci possess a SNAT activity like *S. pseudintermedius* ED99, we tested the ability of 40 different staphylococcal strains belonging to 15 cluster groups to synthesize NAT from TRY as a substrate. HPLC analysis of the bacterial culture supernatants revealed that 22 of the 40 tested strains were able to produce NAT (Table 1). This indicates that SNAT is widespread in the genus *Staphylococcus*.

**Figure 6:**
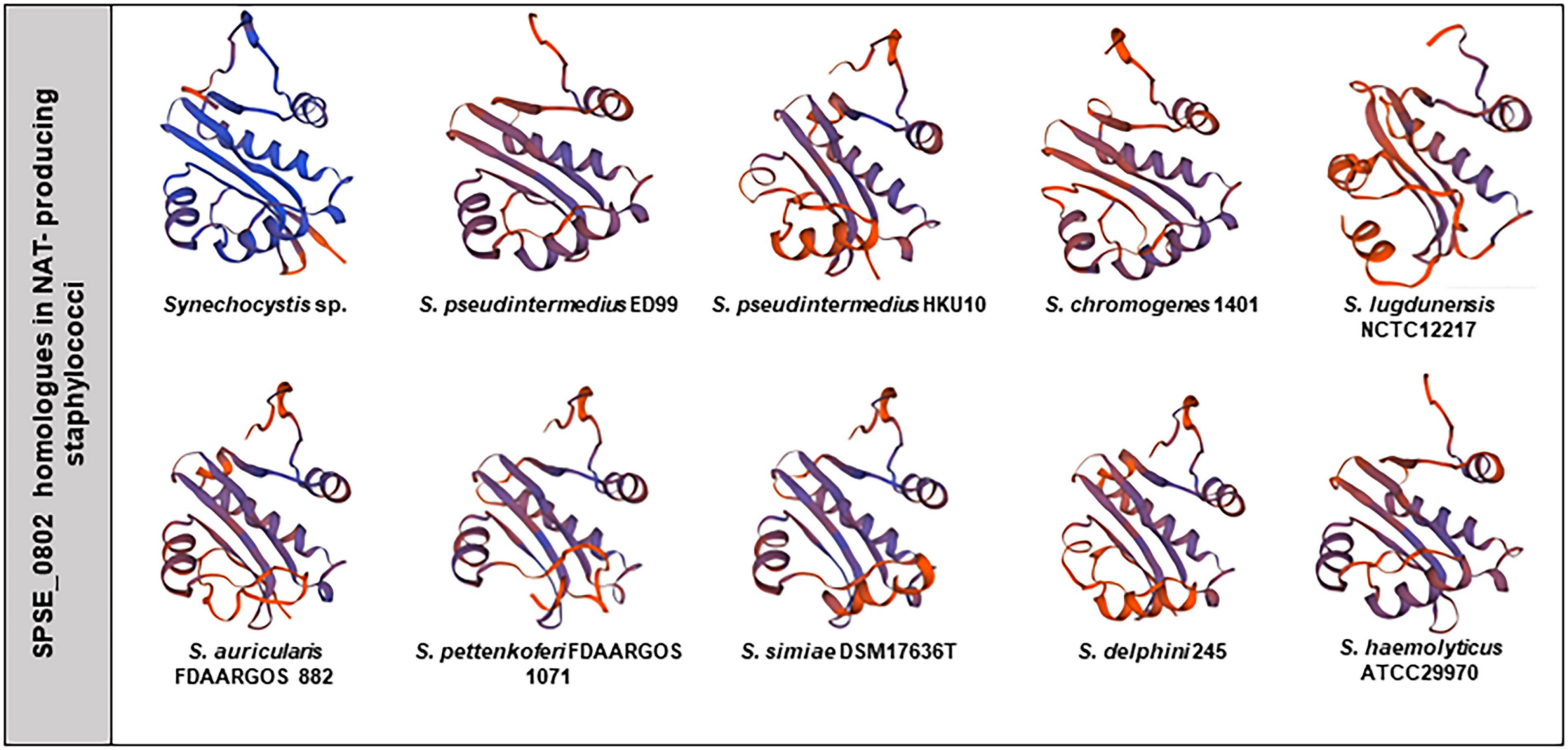
SPSE_0802 protein homologues in staphylococci. Predicted 3D structure of cSNAT, SPSE_0802 and its homologues found in the tested NAT-producing staphylococcal species. The 3D structures were modeled using SWISS-MODEL (44).

As many of the tested staphylococcal strains could produce NAT, we searched for SPSE_0802 homologues in the genus *Staphylococcus* and found 13 hits based on amino acid sequence availability on NCBI. Among the 13 hits, 9 were tested as NAT producers in our experiment. The homologous proteins and accession numbers are listed in Table 2. The predicted 3D structures of the homologues found in NAT-producing strains are presented with that of cSNAT in Figure 6. We note that cSNAT and the homologues in staphylococci share the previously described organization of β -helices and α -sheets. The protein alignment of the SPSE_0802 homologues and the organization of their corresponding genes are presented in Supplementary Figures S2, S3, respectively.

**Table 2:** List of SPSE_0802 protein homologues present in other staphylococcal species, their accession number and their percentage of similarity to SPSE_0802.

Staphylococcal species	SNAT activity	Protein tag	Protein size (aa)	Accession number	Similarity to SPSE 0802 (%)
*S. pseudintermedius* ED99	+	SPSE 0802	140	ADX76117.1	−
*S. lloydii* 2327LY	ND	ISP08_09495	139	QPM74569.1	85
*S. haemolyticus* ATCC29970	+	EQ029_10910	139	QCY39241.1	77
*S. taiwanensis* NTUHS172	ND	HYI43_10955	139	UDI79051.1	76
*S. agnetis* 12B	ND	DWB91_10855	139	QDW99586.1	67
*S. chromogenes* 1401	+	GJU84_10980	140	QIN27537.1	70
*S. pseudoxylosus* 14AME19	ND	JMB28_12950	139	QRA18016.1	69
*S. delphini* 2451	+	IPU21_02605	138	QUM67412.1	45
*S. pseudintermedius* HKU1003	+	SPSINT_2046	138	ADV06574.1	45
*S. simiae* NCTC13838	+	SAMEA4384339_02208	133	SNV80616.1	47
*S. capitis* BN2	+	FRG19_02560	133	QOX60030.1	49
*S. lugdunensis* NCTC12217	+	NCTC12217_00650	133	SQI89931.1	49
*S. auricularis* FDAARGOS882	+	I6G39_02390	133	QPT06522.1	48
*S. pettenkoferi* FDAARGOS1071	+	I6I28_03895	133	QQC38043.1	47
*S. pseudintermedius* ED99	+	SPSE_1761	142	ADX77015.1	20
*S. pseudintermedius* ED99	+	SPSE_0436	138	ADX75773.1	30

The homologues were found based on prediction by SyntTax. Based on our SNAT activity results, 9 of the listed staphylococcus species, tested positive for NAT production (presented with a + sign) and in four species the SNAT activity was not determined (ND). The proteins SPSE_1761 and SPSE_0436 were also added.

## Discussion

SNATs constitute a universal group of enzymes present in vertebrates, plants, insects, yeast and cyanobacteria (Voisin et al., 1984; Byeon et al., 2013; Kang et al., 2013; Lee et al., 2014). In humans, SNAT is produced as the penultimate enzyme in the classical pathway of melatonin biosynthesis (Klein et al., 1997). New functions of SNAT products are still being discovered, which highlights the importance of these enzymes in fulfilling diverse roles across kingdoms (Coon and Klein, 2006; Zhao et al., 2019). Despite sharing a characteristic protein fold and an acetyl-CoA binding pocket, SNATs show limited primary sequence homology as well as different catalytic residues (De Angelis et al., 1998; Dutnall et al., 1998; Hickman et al., 1999; Dyda et al., 2000; Liao et al., 2021). This variation could be behind the enzyme’s wide substrate range and its involvement in alternative pathways in different species (Tan et al., 2016). In addition, the need to be translocated to different parts and organelles contributes to the difference observed on the N-terminal end of the enzymes (Coon and Klein, 2006). For instance, SNAT in *Oryza sativa* (OsSNAT) possess a long N-terminal sequence (residues 1–83), which corresponds to a chloroplast transit sequence (Kang et al., 2013; Liao et al., 2021). Similarly, the N-terminal residues 1–27 in sheep SNAT have no effect on its activity and most likely function as a signal peptide (Hickman et al., 1999).

A number of SNAT proteins have been recombinantly expressed and purified using a tag fused either to the N- or C-terminal ends (Byeon et al., 2013; Kang et al., 2013; Lee et al., 2014; Back et al., 2021). The catalytically active recombinant SNATs presented a variable activity *in vitro* at a wide range of temperature (25–95°C) and pH (6.5–9.0). This range depends on internal characteristics like the isoelectric point, 3-D structure and stability of the protein (Byeon et al., 2013; Kang et al., 2013). Since we were testing SNAT activity in staphylococci, we conducted all the assays at the human body temperature (37°C), despite previous reports on higher optimal temperatures for SNATS in plants and cyanobacteria (Byeon et al., 2013; Kang et al., 2013; Lee et al., 2014). Our results are conforming with the higher SNAT activity reported at pH 8.0 and the higher specificity for TRY as a substrate (Byeon et al., 2013; Kang et al., 2013; Lee et al., 2014). The limited acetylation activity of SPSE_0802, as in other SNATs is associated with their large spectrum of substrates and the resulting low specific binding to a particular substrate (Back et al., 2016).

We focused in our study on *S. pseudintermedius* ED99, as it produces high amounts of SER and TRY (Luqman et al., 2018). ED99 could produce NAS and NAT from 5-HTP and TRY respectively, and the amount was 5–6-fold less in its congenic Δ*SPSE_0802* mutant. Those results confirm the activity of SPSE_0802 as a SNAT enzyme with similar properties to those characterized in cyanobacteria and plants Supplementary Figures S4, S5. Since SNAT products are usually found in the cell cytoplasm, we presume that they are produced mainly to act inside the cell, scavenging free radicals to protect the cell from oxidative stress (Wölfler et al., 1999; Oxenkrug, 2005; Álvarez-Diduk et al., 2015).

Next, we checked for the production of N-acetylated products by different staphylococci using TRY as a substrate. Unlike SER, TRY diffuses into the cell and hence, could be used to test SNAT activity in SadA non-producing strains as well. NAT was detected in the supernatant of ED99 as well as other staphylococcal species (Table 3). Inspection of the genomic locus of *SPSE_0802* homologues revealed that despite their occurrence in many different staphylococcal strains, the upstream and downstream regions seem not to be conserved among the species (Supplementary Figure S3). This suggests horizontal gene transfer of *SPSE_0802*, which was also observed for *sadA* (Luqman et al., 2018).

**Table 3:** Biosynthesis of NAT in staphylococci.

Cluster group	Strain	SNAT activity
Auricularis	*S. auricularis* DSM20609T	+
Muscae	*S. muscae* DSM70687	−
Hyicus	*S. chromogenes* DSM20454T	+
	*S. hyicus* NCTC10350	−
	*S. felis* DSM7377T	−
Intermedius	*S. intermedius* CCM5739	+
	*S. pseudintermedius* ED99	+
	*S. delphini* DSM220771T	+
	*S. schleiferi subsp. schleiferi* DSMZ4	+
	*S. lutrae* DSM10244T	+
Epidemidis	*S. caprae* DSM20608T	−
	*S. saccharolyticus* DSM20359T	−
	*S. capitis* LK499	−
	*S. epidermidis* O47	+
Aureus	*S. aureus* HG003	+
	S. simiae DSM17636T	+
Warneri	*S. wameri* DSM20316T	−
	*S. pasteuri* ATCC51129	−
Haemlolyticus	*S. haemolyticus* CCM2737	+
	*S. hominis* DSM20328	+
Lugdunensis	*S. lugdunensis* ATCCA3809	+
Cohnii-Napelensis	*S. cohnii* DSM20260	+
	*S. nepalensis* DSM15150T	+
Saprophyticus	*S. saprophyticus* NT219	+
	*S. equorum* LTH5155	−
	*S. gallinarum* DSM20610T	−
	S. xylosus DSM20266	−
	*S. succinus* LTH6218/3	+
Arlettae-Kloosi	*S. arlettae* DSM20672T	+
	*S. kloosi* DSM202676T	+
Pettenkoferi-Masilliensis	*S. pettenkoferi* B3117	+
Simulans-Carnosus	*S. simulans* MK148	−
	*S. carnosus* TM300	+
	*S. piscifermentas* LTH3588	+
	*S. condiment* LTH5866	−
Sciuri	*S. sciuri* SC116	−
	*S. vitulinus* DSM 15615 T	−
	*S. fleuretti* DSM13212T	−
	*S. pulvereri* DSM9931	−
	*S. lentus*	−

N-acetyltryptamine production by different staphylococcal laboratory strains was analyzed by HPLC. Resting bacterial cells were fed with 5 mM tryptophan and incubated overnight at 37°C. 22 out of the 40 strains tested positive for NAT production.

SER and TRY are implicated in bidirectional signaling across species. For instance, gut bacteria in humans regulate the production of SER in the intestinal epithelium and lumen, and thereby modulate host physiology in terms of gastrointestinal motility and blood platelet function (Reigstad et al., 2015; Yano et al., 2015). Inversely, high levels of SER improve the fitness of spore-forming bacteria colonizing the GI tract. This effect was found to be mediated by signaling *via* SER, which is imported by those bacteria through a serotonin transporter (SERT), homologous to mammalian SERT (Fung et al., 2019). A similar bidirectional signaling has also been described for the interaction between the human skin and TA-producing bacteria. TAs boost the adherence and internalization of *S. pseudintermedius* ED99 into human colon epithelial cells *via* α2 adrenergic receptors signaling. This renders the bacteria invisible to the immune system. In contrast, TAs benefit the colonized host by acting as β2 adrenergic receptors antagonists on the skin and accelerating wound-healing. Regulating the levels of SER and TRY is hence crucial and can be achieved by different strategies including N-acetylation into NAS and NAT, a modification that redirects them into other signaling pathways.

**Figure 7:**
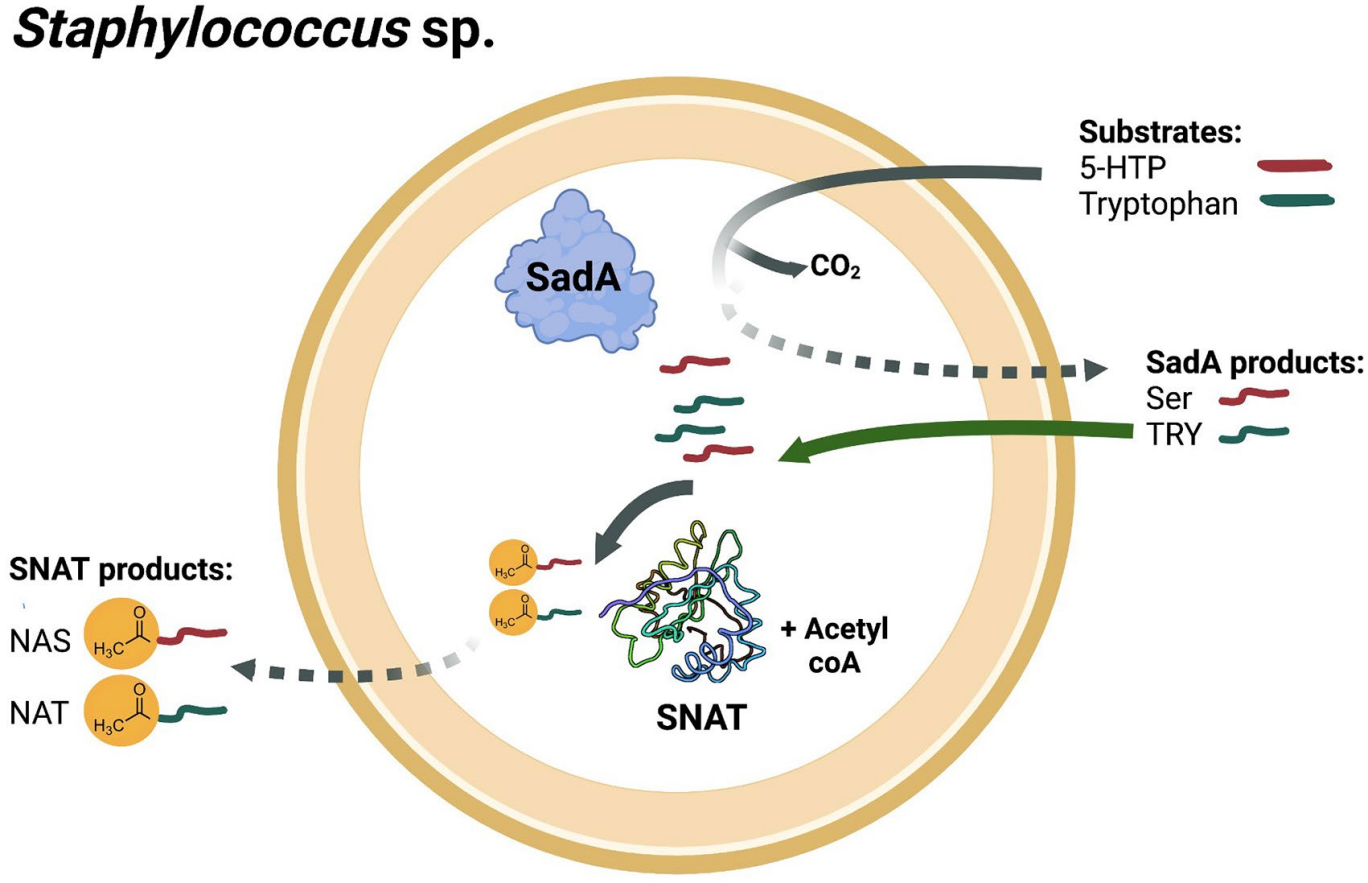
Biosynthesis of NAS and NAT by stahylococci. *Staphylococcus* species harboring SadA and SPSE_0802 could produce NAS from 5-HTP as a substrate in two reactions: decarboxylation of 5-HTP into SER by SadA and N-acetylation of SER into NAS by SPSE_0802. Similarly, staphylococci could synthesize NAT from tryptophan in two reactions: decarboxylation of tryptophan into tryptamine (TRY) by SadA and N-acetylation of TRY into NAT by SPSE_0802. *Staphylococci* that harbor SPSE_0802 but not SadA could also produce NAT when TRY is available in the environment. SadA- and SPSE_0802- products could be detected in the extracellular medium.

Members of the genus *Staphylococcus* are common colonizers of the nares, skin and intestine in humans as well as other mammals (Acton et al., 2009; Parlet et al., 2019; Luqman et al., 2020b; Carroll et al., 2021). Their proximity to the host might have resulted in their acquisition of the *sadA* gene by horizontal gene transfer, enabling them to produce SER and TRY fulfilling different functions (Luqman et al., 2018). Here, we provide evidence that staphylococcal species harbor another gene, namely *SPSE_0802*, that is responsible for catalyzing NAS and NAT biosynthesis. These products are produced in the cytoplasm and function mainly as antioxidants inside the cell nevertheless, they could also be detected in the culture medium. This implies that they could be involved in other processes that affect neurotransmission pathways and play another role in signaling with the colonized host (Figure 7).

## Material and method

### Bacterial strains and growth conditions

Bacterial strains and plasmids used in this study are listed in Supplementary Table S1. For cloning procedures in *S. pseudintermedius ED99*, the genome sequence with GenBank accession number NZ_CP065921.1 was used as a reference. *S. pseudintermedius* strains were grown in tryptic soy broth (TSB) and *E. coli* strains were grown in Luria Bertani (LB) medium. Bacteria were cultivated aerobically (200 rpm) at 37°C. Each experiment was started from an overnight preculture by adjusting the OD_578nm_ to 0.05–0.1 in the corresponding medium. The medium was supplemented with the following antibiotics, where applicable, at the indicated final concentrations: chloramphenicol at 10 μgml−1 for staphylococcal strains and 30 μgml−1 kanamycin for *E. coli* strains.

### BLAST analysis of serotonin N-acetyltransferase of *Synechocystis* sp. against *Staphylococcus pseudintermedius* ED99

The protein sequence of serotonin N-acetyltransferase of *Synechocystis* sp. (Byeon et al., 2013) (GenBank accession number WP_010873901.1) was used to search the homologous protein in *S. pseudintermedius* ED99 using BLASTP. The protein hits were identified as putative SNAT enzymes.

### Bacterial expression and purification of recombinant SNAT candidates

Expression plasmids were constructed using pET28a (Novagen). pET28a was linearized, using the restriction enzymes NotI and NcoI to add a histidine tag to the C-terminal end, and ligated with either of the 3 genes encoding SPSE_1761, SPSE_0802 or SPSE_0436 amplified from *S. pseudintermedius* ED99 genome (oligonucleotides listed in Supplementary Table S2). The ligations were performed using Hi-Fi DNA Assembly Master Mix (New England Biolabs), then transformed into *E. coli* DC10B by heat shock method. The colonies grown on selective agar media containing kanamycin were confirmed by plasmid isolation and sequencing. The plasmids were then transformed into the expression host *E. coli* BL21 (DE3). The clones containing the correct plasmid were cultured overnight at 25°C in LB supplemented with kanamycin. The cells were harvested and lysed using 0.1 mm glass beads in FastPrep instrument (MP Biomedicals). The cell lysate was centrifuged (15,000 *g*, 30 min, 4°C) and the proteins in the supernatant were subjected to protein purification using Ni-NTA superflow resin (IBA). The expression and purification steps were verified by 14% SDS-PAGE. The protein concentration was determined by the Bradford method using a protein assay dye (Bio-Rad, Hercules, CA, United States).

### *In vitro* enzymatic assay

The purified proteins were subjected to *in vitro* enzymatic assay. The purified recombinant proteins (200 μg) were incubated in a total volume of 1 mL containing 1 mM substrate and 1 mM acetyl-CoA in 50 mM potassium phosphate (pH 6.8 or 8) at 37°C. The assay was performed using serotonin as substrate as well as dopamine, tryptamine, phenethylamine, and tyramine. At the indicated time points, the reaction was stopped by adding 250 μL methanol, and the samples were stored at −20°C before being analyzed by HPLC. For the heat inactivation of SPSE_0802, the recombinant protein was incubated at 95°C for 10 min before being added to the reaction mixture.

### HPLC analysis

The *in vitro* enzymatic assays and the bacterial supernatants were analyzed using reversed-phase HPLC (RP-HPLC) as previously described (Luqman et al., 2020a; Luqman et al., 2020). Briefly, the HPLC analysis was performed at room temperature with an Eclipse XDB-C18 column (4.6150 mm; 5 m) (Agilent) and an analytical guard column for Eclipse XDB-C-18 (4.6 12.5 mm; 5 m) (Agilent) with a 15 min linear gradient of 0.1 percent phosphoric acid to acetonitrile and 5 min post time washing with an injection flow rate of 1.5 mL/min, and a sample volume of 10 μL. As a reference, diode array detectors (DAD) were employed at 210 and 360 nm.

### Construction of deletion mutant

The null mutant lacking the putative serotonin N-acetyltransferase-encoding gene in *S. pseudintermedius* ED99 was constructed using the plasmid pBASE6 (Geiger et al., 2012). Briefly, pBASE6 was linearized using EcoRV and ~ 1,000 bp upstream and ~ 1,000 bp downstream fragments of the gene of interest were amplified from the genomic DNA of *S. pseudintermedius* ED99. The fragments were fused by Gibson Assembly (Gibson et al., 2009) using Hi-Fi DNA Assembly Master Mix (New England Biolabs). The resulting plasmid was first introduced into *E. coli* DC10B (Monk et al., 2012) and then into ED99. The deletion mutant construction was performed as previously described (Bae and Schneewind, 2006). Deletion of the genes were confirmed by PCR and sequence analysis. The mutants were named as described in the Supplementary Table S1. Oligonucleotides used are listed in the Supplementary Table S2.

### NAS production by *Staphylococcus pseudintermedius* ED99

HPLC analysis of bacterial cell lysates was performed to determine the production of NAS by *S. pseudintermedius* ED99. The strains were cultured in 10 mL TSB supplemented with 5 mM 5-hydroxytryptophan (5-HTP) and incubated overnight at 37°C with shaking at 150 rpm. Then, the cells were harvested, resuspended in 2 mL potassium phosphate buffer (pH 7.2) and lysed using 0.1 mm glass beads in FastPrep instrument (MP Biomedicals). The cell lysate was centrifuged for 30 min, 5,000 × *g*, 4°C. The supernatant was collected and stored at −20°C until analyzed by HPLC.

### NAT production screening in *Staphylococcus* strains

HPLC analysis of bacterial cultures supernatants was performed to determine the production of NAT by different *Staphylococcus* strains. The strains were cultured in TSB overnight. Then, the cells were harvested, washed with PBS (pH 7.2), and resuspended in PBS (pH 7.2) supplemented with 1% glucose and 5 mM TRY and incubated overnight at 37°C with shaking at 150 rpm; the cell density was kept relatively high (OD_578_ = 50). The cultures were then analyzed using HPLC to determine the presence of NAT.

### Statistical significance

For each experiment, the results were expressed as the mean value ± SEM of data from 3 to 5 replicates. All the statistical analyses were performed using GraphPad Prism software, and a *p*-value of <0.05 was considered statistically significant. Statistical test choice and significance are indicated in the figure legends.

## Data availability statement

The original contributions presented in the study are included in the article/Supplementary material, further inquiries can be directed to the corresponding authors.

## Author contributions

AL and FG conceived the idea. AL and NH designed the experiments. AL, NH, and NL performed all the experiments. All authors contributed to the article and approved the submitted version.

## Funding

This work was supported by the DFG, German Research Foundation, Germany’s Excellence Strategy—EXC 2124—390838134 ‘Controlling Microbes to Fight Infections’ and Institut Teknologi Sepuluh Nopember under project scheme of the Publication Writing and IPR Incentive Program (PPHKI).

## Conflict of interest

The authors declare that the research was conducted in the absence of any commercial or financial relationships that could be construed as a potential conflict of interest.

## Publisher’s note

All claims expressed in this article are solely those of the authors and do not necessarily represent those of their affiliated organizations, or those of the publisher, the editors and the reviewers. Any product that may be evaluated in this article, or claim that may be made by its manufacturer, is not guaranteed or endorsed by the publisher.

## Supplementary material

SUPPLEMENTARY FIGURE S1 Standard curves for quantification of NAS and NAT by HPLC analysis.

SUPPLEMENTARY FIGURE S2 Protein alignment of SPSE_0802 homologues in staphylococci. The alignment of putative SNAT in staphylococcal strains was performed using Jalview. The consensus residues and their degree of conservation are noted.

SUPPLEMENTARY FIGURE S3 Comparison of gene location of SPSE_0802 homologues in some NAS-producing staphylococci. Putative SNAT-encoding genes in staphylococcal strains were predicted using SyntTax.

SUPPLEMENTARY FIGURE S4 Phylogenetic tree of SPSE_0802 and other SNATs from plants and cyanobacteria. The phylogenetic tree was built based on the BLASTP results of SPSE_0802 outside the genus Staphylococcus. The hits were checked for the amino acid sequence on NCBI and were aligned. The multiple sequence alignment was used to construct the phylogenetic tree using maximum likelihood (ML) analysis in Mega11.

SUPPLEMENTARY FIGURE S5 Multiple sequence alignment of SPSE_0802 and the homologous SNATs in plants and cyanobacteria.

## Abbreviations

GNAT: GCN5-related N-acetyltransferases
NAS: N-acetylserotonin
NAT: N-acetyltryptamine
SadA: enzyme of staphylococcal aromatic amino acid decarboxylase
SER: serotonin or 5-hydroxytryptophan (5-HTP)
SNAT: serotonin N-acetyltransferase
TA: trace amines (PEA, TRY, TYM)
TRY: tryptamine
