# Supplementary material for: Identification of a serotonin N-acetyltransferase from Staphylococcus pseudintermedius ED99
Source: Front Microbiol. 2023 Feb 22;14:1073539. doi: 10.3389/fmicb.2023.1073539 (PMC9992809; doi:10.3389/fmicb.2023.1073539)
Supplement: SUPPLEMENTARY FIGURE S1 — Standard curves for quantification of NAS and NAT by HPLC analysis. [file Data_Sheet_1.zip › Table S1.docx]

**Table S1. Strains and plasmids used in this study. In addition to the staphylococcal strains listed in Table 2.**

| **Strain** | **Description** | **Reference** |
| --- | --- | --- |
| **Staphylococcus** | | |
| *S. pseudintermedius* ED99 | Canine bacterial pyoderma clinical isolate | (2) |
| RN4220 | Restriction-deficient *S. aureus* strain | (3) |
| *S. pseudintermedius* ED99 Δ*SPSE_0802* | Markerless deletion of *SPSE_0802* | This study |
| ***E. coli*** | | |
| *E. coli* DC10B | Common laboratory strain for cloning | (4) |
| *E. coli* BL21(DE3) | Common protein expression strain | (5) |
| **Plasmids** | | |
| pBASE6 | Temperature-sensitive plasmid used for knockout | (6) |
| pBASE-Δ*SPSE_0802* | Plasmid for markerless deletion of *SPSE_0802* | This study |
| pET28a | IPTG-inducible expression for *E. coli* | Novagen |
| pET28a-SPSE_0802-his | IPTG-inducible expression of his tagged SPSE_0802 | This study |
| pET28a-SPSE_0436-his | IPTG-inducible expression of his tagged SPSE_0436 | This study |
| pET28a-SPSE_1761-his | IPTG-inducible expression of his tagged SPSE_1761 | This study |
