# Supplementary material for: Identification of a serotonin N-acetyltransferase from Staphylococcus pseudintermedius ED99
Source: Front Microbiol. 2023 Feb 22;14:1073539. doi: 10.3389/fmicb.2023.1073539 (PMC9992809; doi:10.3389/fmicb.2023.1073539)
Supplement: SUPPLEMENTARY FIGURE S1 — Standard curves for quantification of NAS and NAT by HPLC analysis. [file Data_Sheet_1.zip › Table S2.docx]

**Table S2. Oligonucleotides used in this study**

| **Primer** | **Sequence (5’-3’)** |
| --- | --- |
| **SPSE0802_fwd** | AGAGTTCGAGGAGGTTTAATATGAATATAAAACTTGTTAACACATTT |
| **SPSE0802_rev** | TTAAGTACTTCAGCTAATTATTTTTCAAATTGTGGATGTGACCATGATTCAGTATATTCGATTTTCAATTTTTC |
| **SPSE0436_fwd** | AGAGTTCGAGGAGGTTTAATATGACAACTGAAGTAAAATATGAAATTC |
| **SPSE436_rev** | TTAAGTACTTCAGCTAATTATTTTTCAAATTGTGGATGTGACCACTTTTCAACGGGATAGTG |
| **SPSE1761_fwd** | AGAGTTCGAGGAGGTTTAATATGATTAAAACAGTAGAAACGG |
| **SPSE1761_rev** | TTAAGTACTTCAGCTAATTATTATTTTTCAAATTGTGGATGTGACCAAGAAGTGAGGTGCTTTTC |
| **SPSE0802 up fwd** | CGCGCAGATCTGTCGACGATAACACAGAACTAGAAGTTG |
| **SPSE0802 up rev** | TATTCGATTTCAAGTTTTATATTCATACTAGGC |
| **SPSE0802 down fwd** | ATAAAACTTGAAATCGAATATACTGAATCATAGTTTGAC |
| **SPSE0802 down rev** | \| TGCAGGCATGCAAGCTTGATAGTTGGCGC \|  \| \| --- \| --- \| |
