# Supplementary figures and images for: Identification of a serotonin N-acetyltransferase from Staphylococcus pseudintermedius ED99
Source: Front Microbiol. 2023 Feb 22;14:1073539. doi: 10.3389/fmicb.2023.1073539 (PMC9992809; doi:10.3389/fmicb.2023.1073539)

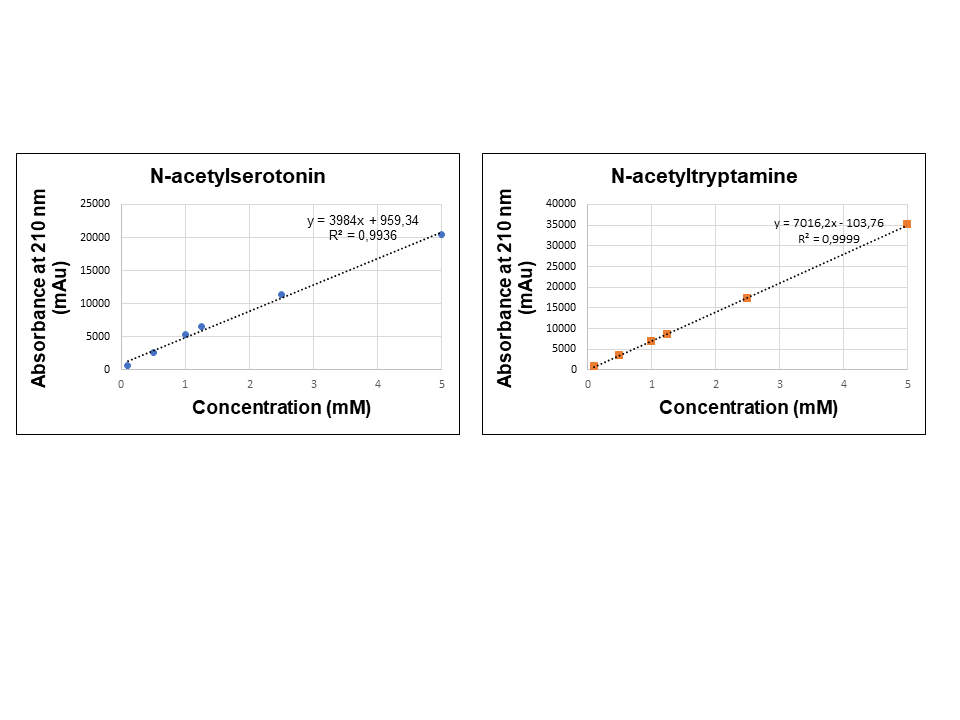

Supplement: SUPPLEMENTARY FIGURE S1 — Standard curves for quantification of NAS and NAT by HPLC analysis. [file Data_Sheet_1.zip › Image 1.TIF]

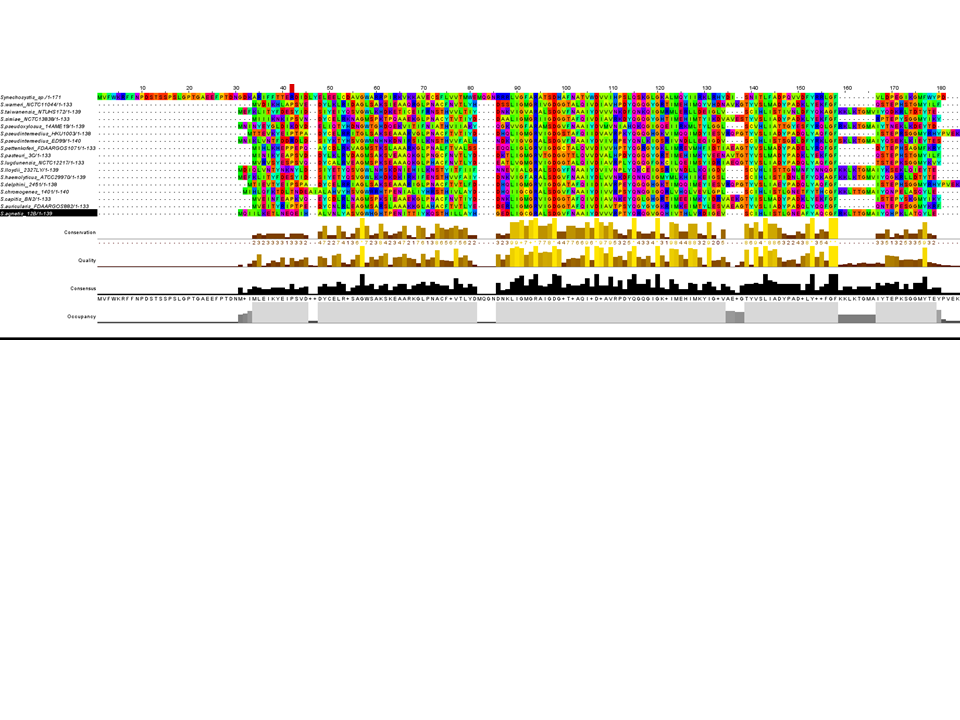

Supplement: SUPPLEMENTARY FIGURE S1 — Standard curves for quantification of NAS and NAT by HPLC analysis. [file Data_Sheet_1.zip › Image 2.TIF]

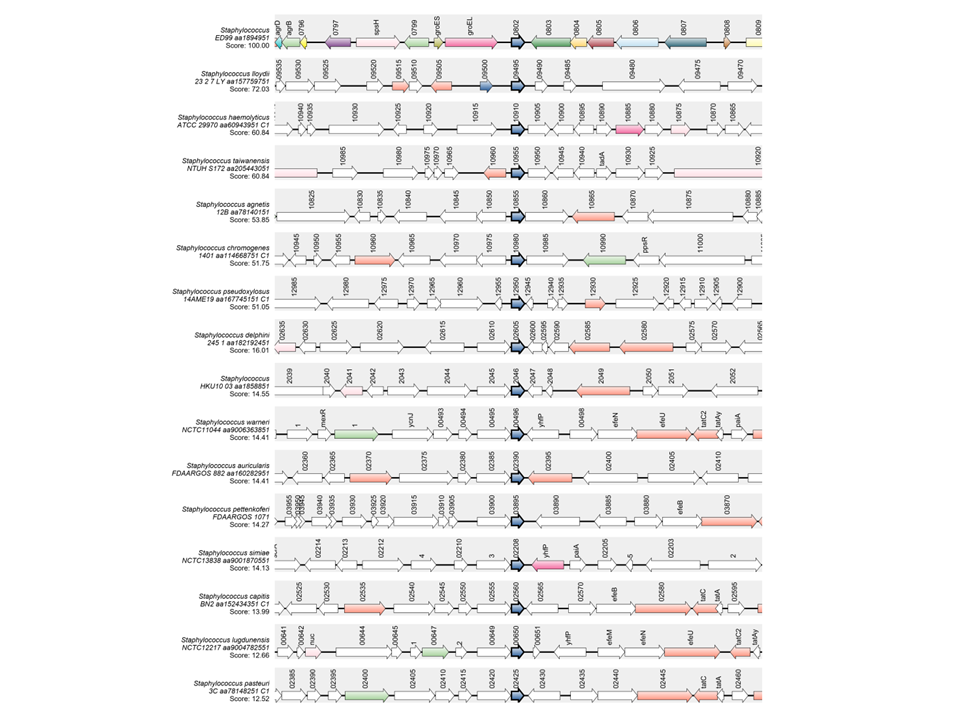

Supplement: SUPPLEMENTARY FIGURE S1 — Standard curves for quantification of NAS and NAT by HPLC analysis. [file Data_Sheet_1.zip › Image 3.TIF]

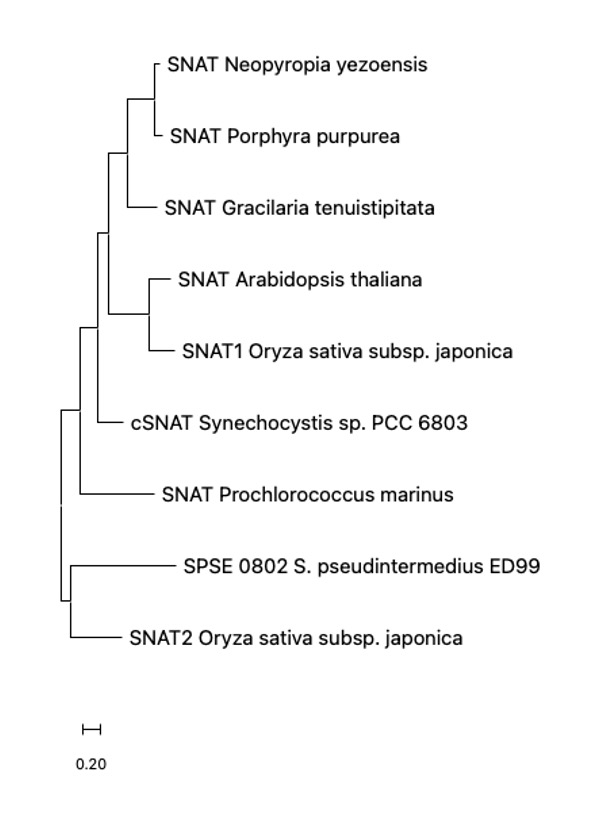

Supplement: SUPPLEMENTARY FIGURE S1 — Standard curves for quantification of NAS and NAT by HPLC analysis. [file Data_Sheet_1.zip › Image 4.JPEG]

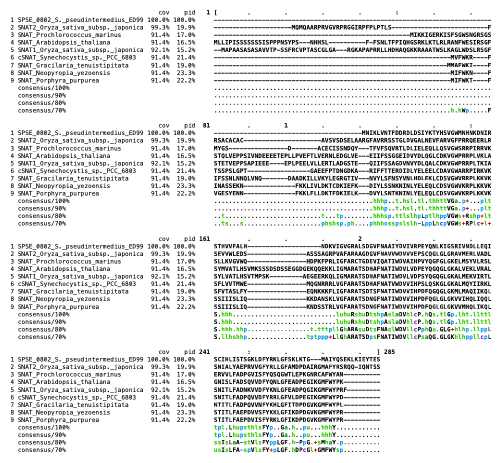

Supplement: SUPPLEMENTARY FIGURE S1 — Standard curves for quantification of NAS and NAT by HPLC analysis. [file Data_Sheet_1.zip › Image 5.PNG]
